# Role of Sox9 in BPD and its effects on the Wnt/β-catenin pathway and AEC-II differentiation

**DOI:** 10.1038/s41420-023-01795-2

**Published:** 2024-01-11

**Authors:** Di Wu, Dongqin Bai, Miao Yang, Bo Wu, Wei Xu

**Affiliations:** 1 Department of Pediatrics, Shengjing Hospital of China Medical University, Shenyang, China; 2 Department of Intensive Care unit, The Sixth Affiliated Hospital of Harbin Medical University, Harbin, China

**Keywords:** Transdifferentiation, Disease model, Respiratory tract diseases

## Abstract

The excessive activation of the Wnt/β-catenin signaling pathway is an important regulatory mechanism that underlies the excessive proliferation and impaired differentiation of type 2 alveolar epithelial cells (AEC-II) in bronchopulmonary dysplasia (BPD). Sox9 has been shown to be an important repressor of the Wnt/β-catenin signaling pathway and plays an important regulatory role in various pathophysiological processes. We found that the increased expression of Sox9 in the early stages of BPD could downregulate the expression of β-catenin and promote the differentiation of AEC-II cells into AEC-I, thereby alleviating the pathological changes in BPD. The expression of Sox9 in BPD is regulated by long noncoding RNA growth arrest-specific 5. These findings may provide new targets for the early intervention of BPD.

## Introduction

Pulmonary organogenesis requires precise timing and coordination to influence the spatial organization and function of parenchymal cells. Owing to exposure to hyperoxia and inflammation, the development of the lungs in preterm infants born during the cystic phase of lung development may be permanently altered, resulting in persistent structural damage and lifelong respiratory impairment, which can lead to bronchopulmonary dysplasia (BPD) [1, 2]. Hyperoxia can induce the BPD phenotype and alter the composition of cellular compartments, especially alveolar epithelia, stromal fibroblasts, capillary endothelia, and the macrophage population [3]. The abnormal ability of type 2 alveolar epithelial cells (AEC-II) to proliferate and differentiate into AEC-I is an important factor in this pathological change.

Animal experiments have shown that in the pathogenesis of BPD, various signaling factors, such as transforming growth factor β, connective tissue growth factor, fibroblast growth factor 10, vascular endothelial growth factor, caveolin-1, wingless and int-1 (WNT)/β-catenin, and elastin, are abnormally regulated [4]. The WNT signaling pathway is one of the key signaling pathways involved in the regulation of the stem and progenitor cells of various organs. In addition to its role in embryonic and fetal lung development, the Wnt signaling pathway is crucial to lung homeostasis and regeneration [5]. Our previous studies on the canonical and non-canonical pathways mediated by Wnt3a and Wnt5a have shown that both alveolar epithelial cell damage repair and alveolarization are regulated in hyperoxia-induced lung injury [6] and that Wnt5a is involved in the pathological process of neonatal hyperoxia-induced injury [7].

The activity of the Wnt pathway is regulated by various means, such as the acylation of the Wnt protein, stability of the Wnt receptor, and abundance of the β-catenin protein. Many studies have reported that Sox9 is a transcription factor with strong inhibitory effects on Wnt signaling and β-catenin levels. During embryonic development, Sox9 plays an important role in the regulation of sex determination [8], cartilage development [9], and development of digestive organs [10] through mutual antagonism with the Wnt signaling pathway. During lung development, Sox9 regulates normal branch morphogenesis and lung epithelial development through various signaling pathways, inhibits the premature initiation of alveolarization, and is expressed in adult airway distal progenitor cells and maintains their normal function [11, 12]. However, in some pathological conditions, such as ovarian cancer, endometrial cancer, and non-small cell lung cancer, Sox9 overexpression can abnormally activate the wnt pathway and promote tumorigenesis and progression. Studies have confirmed that the activation of Sox9 is crucial for the recovery of lung function after acute lung injury [13]. However, it is not clear whether Sox9 is also involved in the overactivation of wnt pathway in BPD, thus affecting the development of lung epithelium.

Long noncoding RNAs (lncRNAs) are long-chain RNA molecules that play important roles in many biological functions, including cell growth and developmental processes, by regulating cellular signaling pathways, such as the JAK/STAT, PI3K/Akt, and Wnt signaling pathways [14, 15]. High expression of lncRNA Growth arrest-specific transcript 5 (GAS5) can inhibit cell cycle progression, whereas the downregulation of GAS5 can inhibit cell apoptosis and promote cell division [16]. GAS5 is downregulated in childhood pneumonia, acute lung injury, and asthma, and its overexpression can cause the inhibition of inflammation and epithelial cell apoptosis [17–19]. In our previous genomic study on lipopolysaccharide-induced acute lung injury, the expression of GAS5 was significantly altered [20], but its expression pattern in BPD was unclear. Through a bioinformatics analysis, we found several microRNAs (miRNAs) that coexisted with GAS5 and Sox9, which may constitute a competing endogenous RNA regulatory pathway.

Therefore, we hypothesize that Sox9 in BPD may affect AEC-II differentiation through the Wnt pathway, and its upstream is regulated by GAS5.

## Results

### In BPD, the alveolar structure is simplified, alveolarization is impaired, and expression of Sox9 is increased in the early stage of the disease

**Fig. 1:**
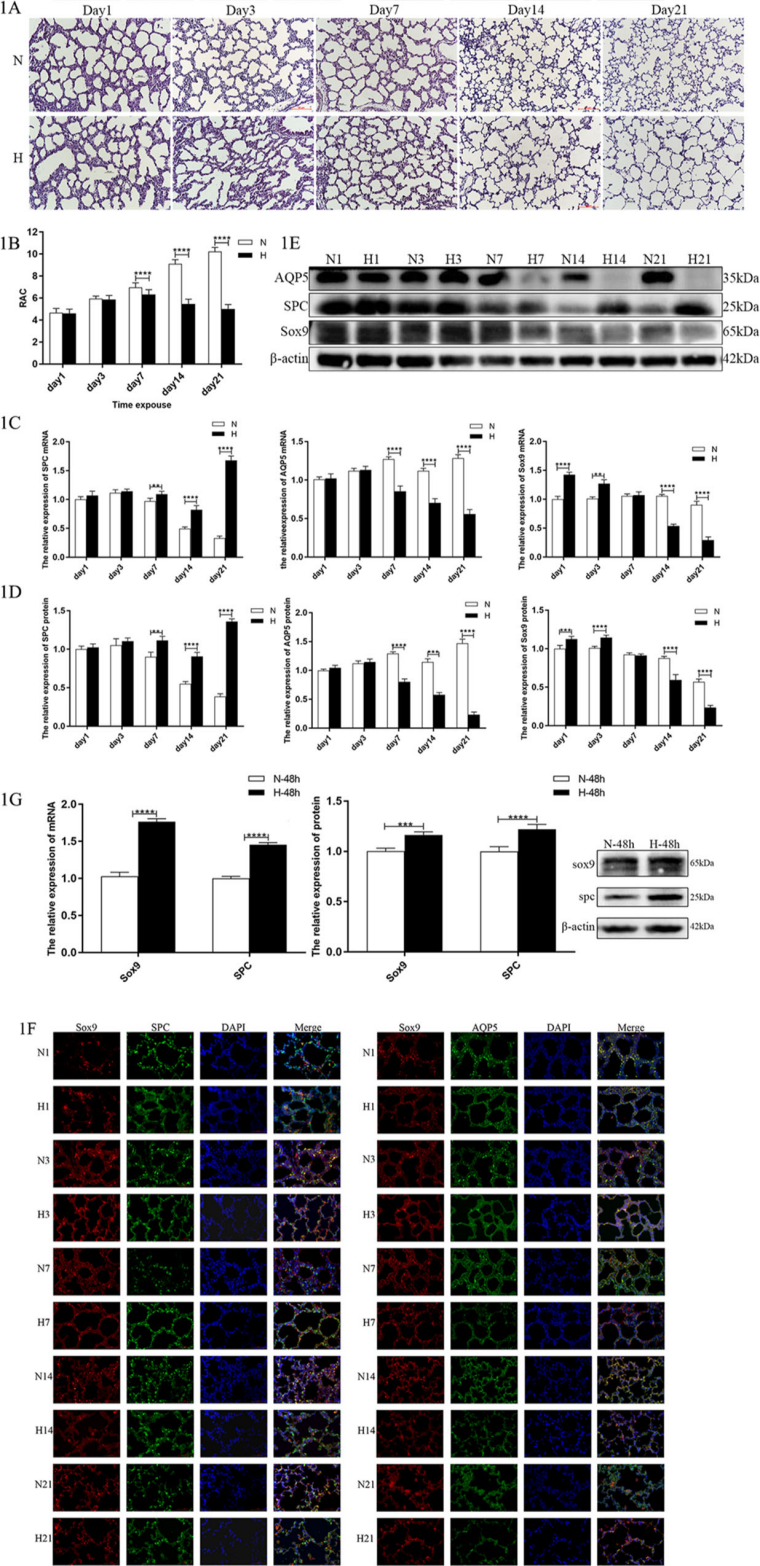
In BPD, alveolation is impaired, and Sox9 expression level is significantly changed. **A** Representative images of H&E staining of lung tissue. Morphology was examined using light microscopy (×200). Scale bar: 50 μm. **B** Comparison of RAC values in lung tissues. **C** Relative levels of SPC, AQP5, Sox9 mRNAs in rat lung tissue (*n* = 6). **D** Relative levels of SPC, AQP5, Sox9 protein in rat lung tissue (*n* = 6). **E** Target band intensity was normalized to β-actin. **F** Paraffin-embedded tissue sections were double stained with Sox9 (red) + AQP5 (green) immunofluorescence (×400). **G** Relative levels of SPC, AQP5, Sox9 mRNAs, and protein in the in vitro cell model. Target band intensity was normalized to β-actin. (**P < 0.01, ***P < 0.005, and ****P < 0.001 compared with the control group).

The hematoxylin and eosin staining of the lung tissue (Fig. 1A) showed that as the rats aged, the hyperoxic model group (H) changed significantly from day 7 onward. The alveoli became enlarged, number of alveoli decreased, alveolar wall thickened, and alveolar crest and secondary septum became less visible or even disappeared. From days 14 to 21, the size of the alveolar cavity increased significantly, alveolar fusion appeared, the number of alveoli was significantly reduced, and the alveolar structure was simplified.

The RAC, which is positively correlated with lung development, refers to the number of alveoli on the vertical line from the center of the respiratory bronchioles to the nearest fibrous septum (or pleura) and is an important indicator for evaluating the maturity of developing lungs. In fig. 1B, the RAC of the control group gradually increased, indicating that the alveoli undergoing alveolarization were gradually maturing. The RAC value of the model group began to decrease significantly on day 7, and the difference was more significant from days 14 to 21 (*P* < 0.001). This indicated that disordered alveolarization is present in BPD.

Then, we detected the expression of the AEC-II surface marker SPC and the AEC-I surface marker AQP5 in animal models. The expression of SPC was increased while AQP5 was decreased. The changes in their expression were significant after 7 days of exposure to hyperoxia (Fig. 1C–E), which confirmed the hyperproliferation of AEC-II cells in BPD and their impaired conversion to AEC-I.

The results of the PCR and western blotting analyses showed that in the early stage of BPD the expression of Sox9 increased (Fig. 1C–E), exhibited a downward trend, and was lower than that in the control group after 7 days.

The immunofluorescence staining of the tissue sections (Fig. 1F) showed that with prolonged hyperoxic exposure, the immunofluorescence intensities of Sox9 and AQP5 gradually weakened, whereas the fluorescence intensity of SPC gradually increased, suggesting that Sox9 may be involved in the regulation of AEC-II cell proliferation and differentiation.

The PCR and western blot results further showed that the expression of SPC and Sox9 increased in the in vitro cell model (Fig. 1G), which was consistent with the early manifestations in the BPD animal model.

### The distribution of Sox9 in the nuclei and cytoplasm of cells changes during the proliferation and transformation of AEC-II cells. The cytoplasmic expression of Sox9 is increased, and nuclear expression of AEC-II cells is decreased in hyperoxic culture

**Fig. 2:**
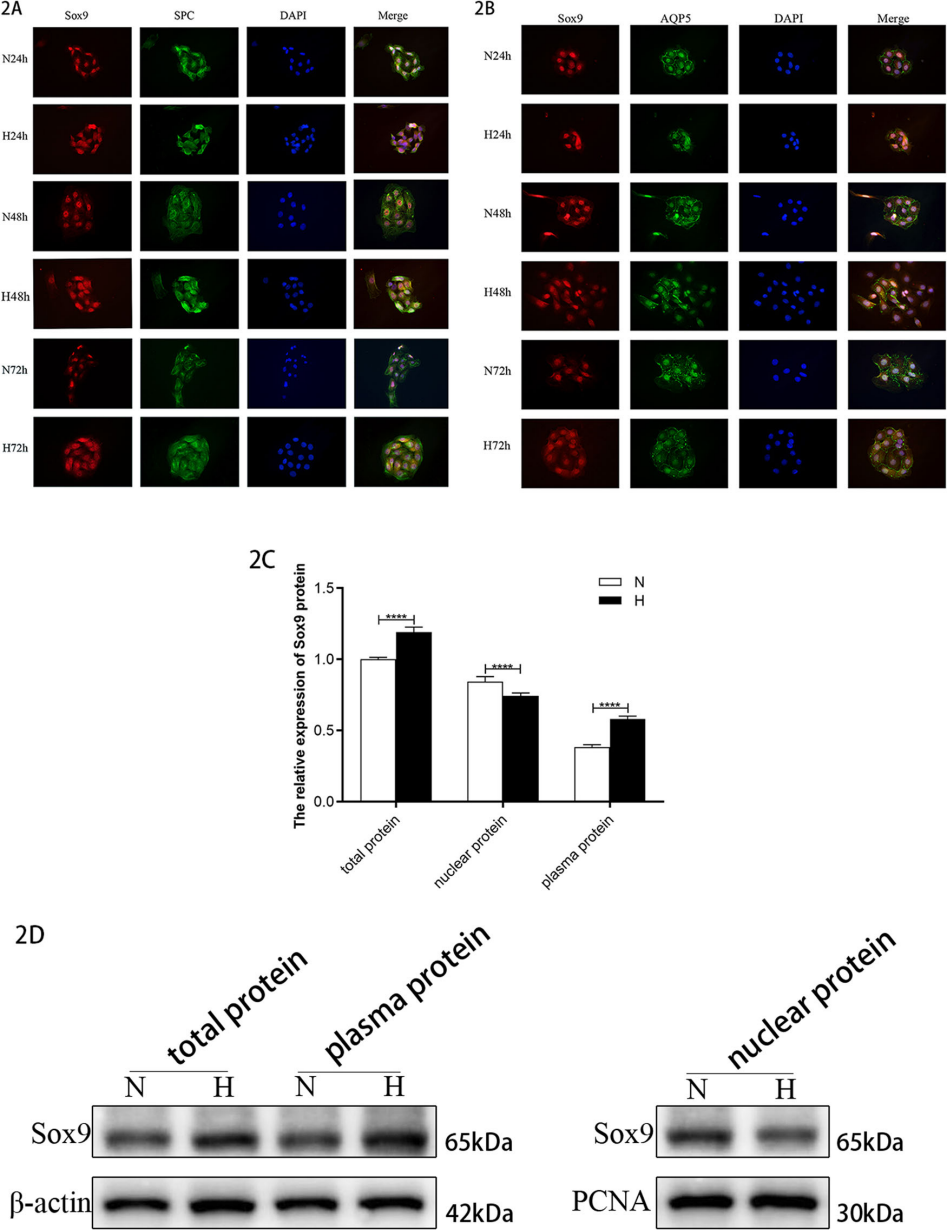
Changes in the expression of Sox9 during primary AEC-II culture and differentiation (×400). **A** Sox9 and SPC immunofluorescence double staining. **B** Sox9 and AQP5 immunofluorescence double staining. **C** Changes in Sox9 protein expression in an in vitro cell model. **D** Total protein and plasma protein target bands were normalized to β-actin, and nucleoprotein target band intensity was normalized to PCNA. (This blot is the cropped image)(****P < 0.001 compared with the control group).

The results of the immunofluorescent double staining of the primary AEC-II cells showed that the expression and fluorescence intensity of SPC gradually decreased during the culture process (Fig. 2A). By contrast, the expression and fluorescence intensity of AQP5 increased (Fig. 2B), suggesting that the primary AEC-II cells had differentiated into AEC-I. Sox9 was expressed in the nuclei of the primary AEC-II cells, and its expression gradually weakened over time. The expression of Sox9 increased in the hyperoxic culture, especially in the cytoplasm; however, the change in its expression in the nucleus was not significant.

The nucleocytoplasmic protein was extracted in vitro for a western blot analysis. The results showed that the nuclear expression of the Sox9 protein in the cells of the model group decreased, whereas its expression in the plasma increased (Fig. 2C, D).

### Exogenous Sox9 can improve alveolarization in animal models of BPD by promoting AEC-II cell proliferation and differentiation into AEC-I

**Fig. 3:**
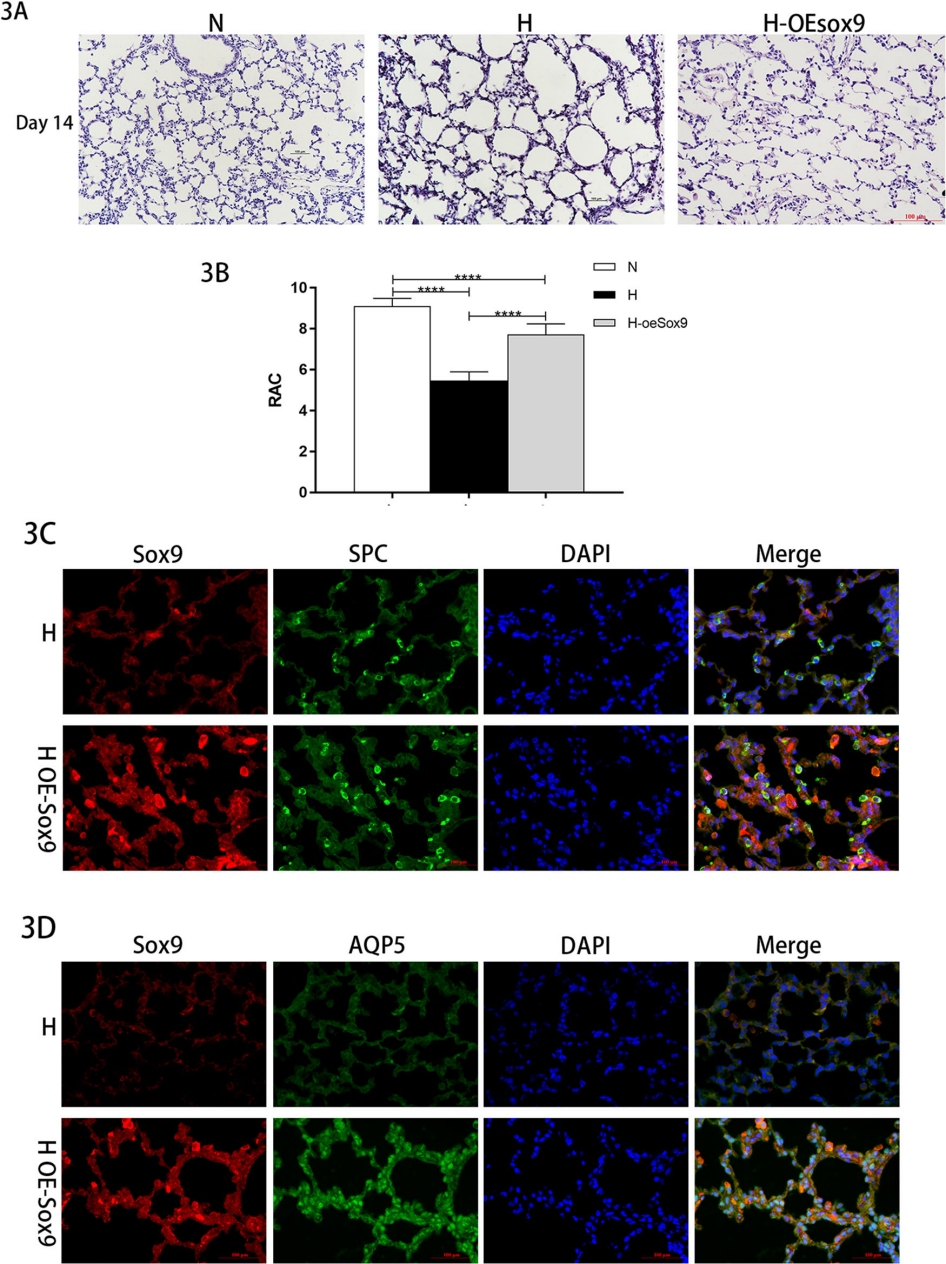
Overexpression of Sox9 in BPD rat models can promote the differentiation of AEC II. **A** Sox9-overexpression improved alveolar development. Scale bar: 50 μm. **B** Comparison of RAC values in lung tissues (****P < 0.001). RAC increased in the overexpression group compared with that in the model group (*n* = 6). **C** Paraffin-embedded tissue sections with Sox9 (red) + SPC (green) immunofluorescence double staining. **D** Paraffin-embedded tissue sections were double stained with Sox9 (red) + AQP5 (green) immunofluorescence.

We administered exogenous Sox9 to animal models of BPD. In the model group, exogenous overexpressed plasmid was injected into the trachea every other day from days 5 to 14. The staining of the lung tissue sections showed that the development of the alveoli in the BPD Sox9-overexpression group was improved compared with that in the BPD model group (Fig. 3A). The number of alveoli increased, their structure became more complete, their size was more uniform, and the RAC value increased (Fig. 3B), which indicated that exogenous Sox9 can improve the degree of alveolarization in BPD lung tissue. The immunofluorescence double staining (Fig. 3C, D) also showed that the number of SPC-labeled cells decreased compared to that in the model group, while the fluorescent expression of AQP5 was enhanced. This confirmed that exogenous Sox9 can promote the proliferation and differentiation of AEC-II cells into AEC-I.

**Fig. 4:**
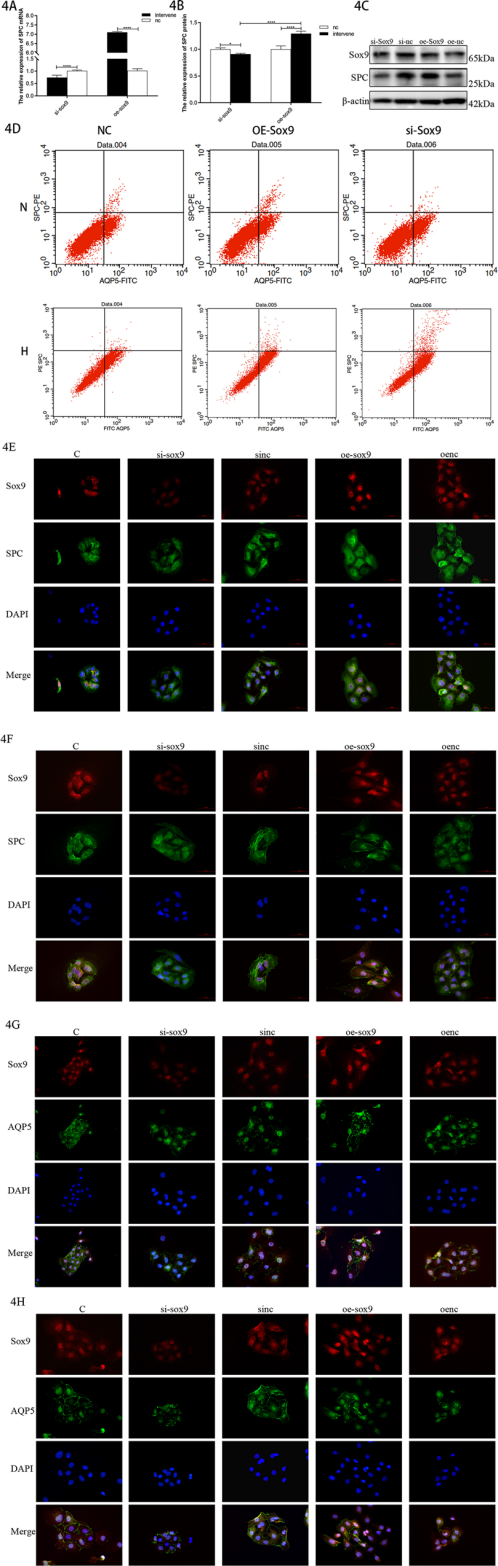
Effect of Sox9 on the proliferation and differentiation of AEC-II cells. **A** Relative levels of SPC mRNAs after Sox9 intervention. **B** Relative levels of SPC protein after Sox9 intervention. **C** Target band intensity is normalized to β-actin. (This blot is the cropped image) (*P < 0.05 and ****P < 0.001 compared with the NC). **D** Flow cytometry analysis of primary cells. **E** Sox9 (red) + SPC (green) immunofluorescence double staining in the control group. **F** Sox9 (red) + SPC (green) immunofluorescence double staining in the model group. **G** Sox9 (red) + AQP5 (green) immunofluorescence double staining in the control group. **H** Sox9 (red) + AQP5 (green) immunofluorescence double staining in the model group.

We further detected the changes in SPC expression after administering Sox9 in an in vitro cell model. The expression of SPC decreased after the knockdown of Sox9 under hyperoxic conditions and increased after the overexpression of Sox9 (Fig. 4A–C). Sox9, therefore, promotes the proliferation of AEC-II cells in BPD.

To verify the effects of Sox9 on AEC-II cell differentiation, we overexpressed Sox9 in primary cells and analyzed the degree of AEC-II cell differentiation using flow cytometry. The results showed that the number of AEC-II cells increased after the overexpression of Sox9 in the control group (Fig. 4D), while the number of AEC-I cells increased in the model group. Double immunofluorescence staining showed that in the model group, SPC expression was increased after the knockdown of Sox9 in the AEC-II cells and decreased after Sox9 had been overexpressed. In addition, the AEC-II cells with overexpressed Sox9 in the model group tended to grow from the typical round and oval-island shape to the divergent polygonal shape of the AEC-I cells (Fig. 4E, F). Compared to the control, in the model group, the AQP5 fluorescence signal of the AEC-I cells overexpressing Sox9 was enhanced, and the morphologies of the cell membranes were clearer and more continuous (Fig. 4G, H). The overexpression of Sox9 may, therefore, promote the rapid differentiation of AEC-II cells into AEC-I under hyperoxia.

### The Wnt/β-catenin pathway is overactivated in BPD, and Sox9 can downregulate the expression of β-catenin and affect its nucleoplasmic distribution

**Fig. 5:**
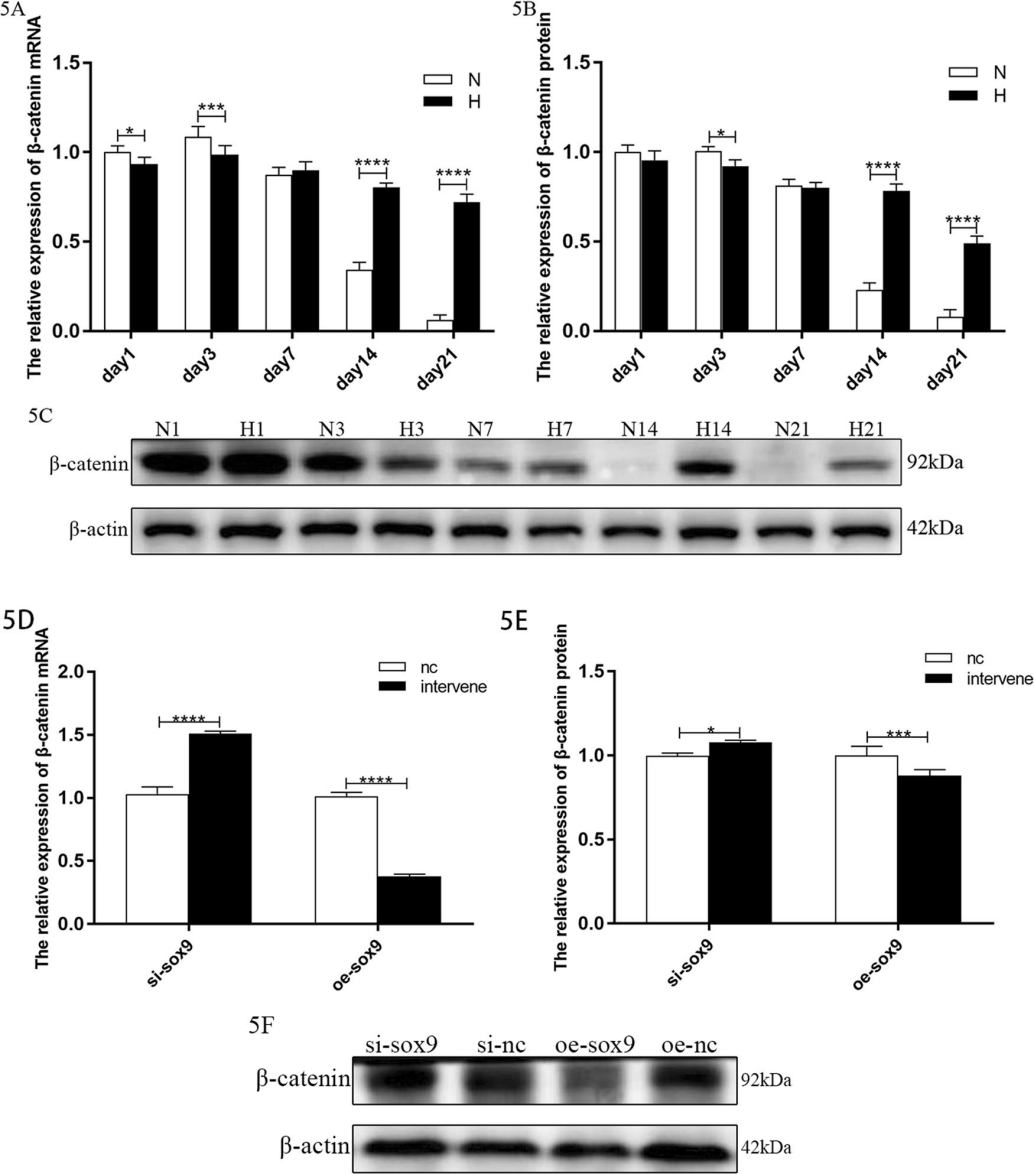
Changes in the expression of β-catenin. **A** Relative levels of β-catenin mRNAs in lung tissues (*n* = 6). **B** Relative levels of β-catenin protein in lung tissues (*n* = 6). **C** Target band intensity was normalized to β-actin. **D** Relative levels of β-catenin mRNAs in cells after Sox9 intervention. **E** Relative levels of β-catenin protein in cells after Sox9 intervention. **F** Target band intensity was normalized to β-actin. (This blot is the cropped image). (*P < 0.05, *** *P* < 0.005, and **** *P* < 0.001 compared with the control group).

Our previous studies showed that the Wnt/β-catenin pathway is overactivated in BPD. In this study, as our previous results, β-catenin exhibited an overall upward trend in the model group compared with that in the control group. Its expression further decreased on days 1–3 and increased after day 7 (Fig. 5A–C).

We further detected the changes in β-catenin expression after the Sox9 intervention in an in vitro cell model. The results showed that the expression of β-catenin increased after knockdown of Sox9 and decreased after overexpression of Sox9 (Fig. 5D–F).

**Fig. 6:**
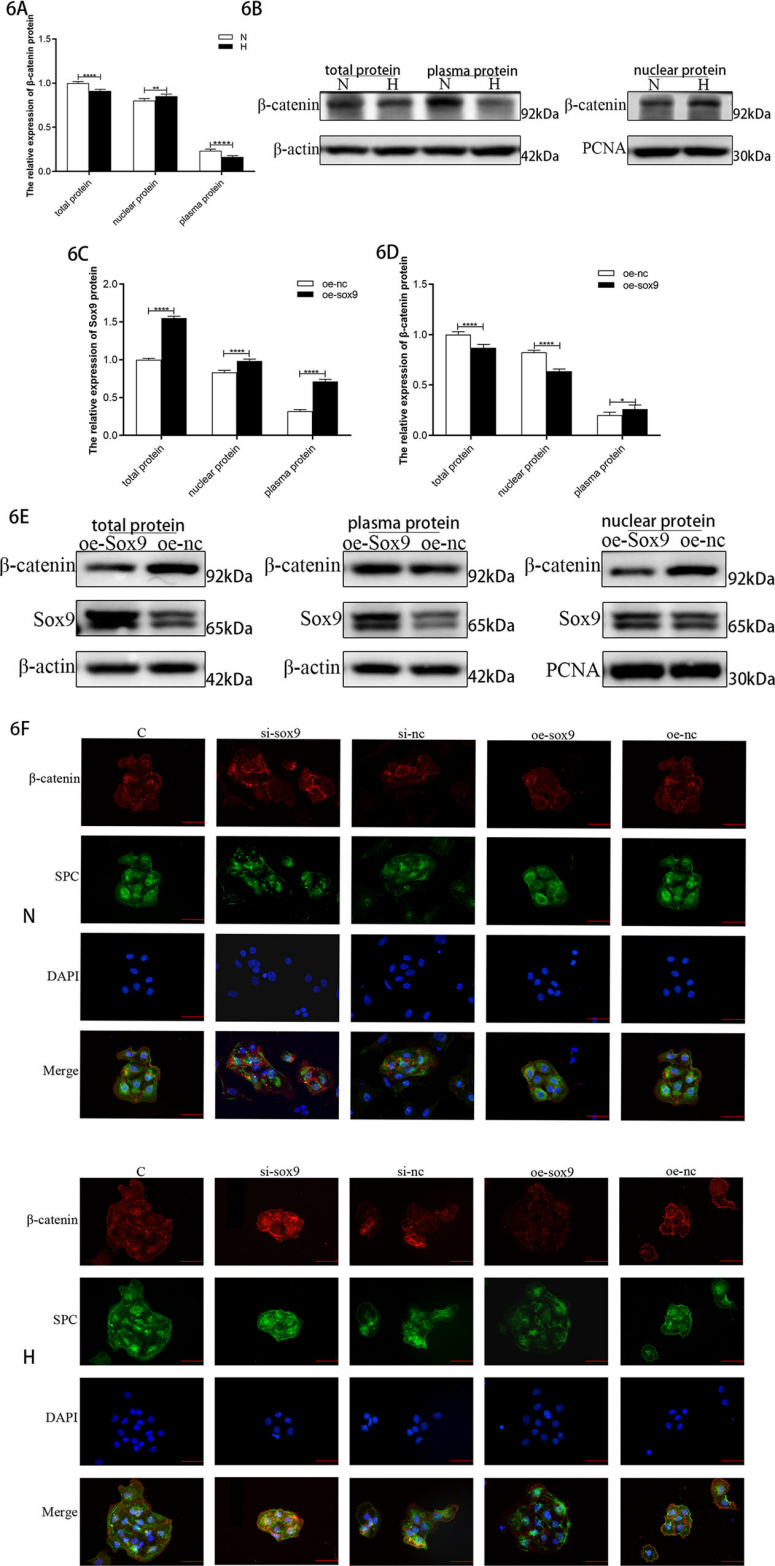
Changes in the distribution of β-catenin in the nucleus and plasma. **A** Relative levels of β-catenin protein in the hyperoxic cell model. **B** The total protein and plasma protein target band intensity was standardized using β-actin, and the nuclear protein target band intensity was standardized using PCNA (This blot is the cropped image). **C** Relative levels of Sox9 protein after the overexpression of Sox9 in the hyperoxic cell model. **D** Relative levels of β-catenin protein after the overexpression of Sox9 in the hyperoxia cell model. **E** Total protein and plasma protein target band intensity was normalized to β-actin, and the nuclear protein target band intensity was normalized to PCNA. (This blot is the cropped image) (*P < 0.05 and ****P < 0.001 compared with the control group). **F** β-Catenin (red) + SPC (green) immunofluorescence double staining in the primary AEC-II cells.

The nuclear and cytoplasmic distribution of β-catenin in the in vitro cell model was examined using western blots. The expression of the total and plasma β-catenin proteins in the cells of the model group decreased, but the nuclear expression of β-catenin increased (Fig. 6A, B). The total protein expression level (in both the nucleus and plasma) increased after overexpression of Sox9 (Fig. 6C), with the increase in the plasma protein expression being more significant. However, the total protein expression of β-catenin decreased, its nuclear protein expression decreased, and its plasma protein expression increased (Fig. 6D, E), which confirmed that the overexpression of Sox9 can reduce the expression of β-catenin in the nucleus.

Next, we intervened Sox9 in primary AEC-II cells and further performed β-catenin + SPC immunofluorescence double staining. The results (Fig. 6F) showed that the fluorescent signal of β-catenin was significantly enhanced after the knockdown of Sox9, while it was weakened after the overexpression of Sox9. Compared with that in the control group, the expression of β-catenin in the nucleus increased after the knockdown of Sox9 in the model group. These results suggest that the overexpression of Sox9 can not only downregulate the overall expression level of β-catenin but also affect the nuclear expression of β-catenin, thereby regulating the activation of the Wnt pathway.

### Sox9 expression in BPD is regulated by the lncRNA GAS5 but not through the miR-1912-3p pathway

**Fig. 7:**
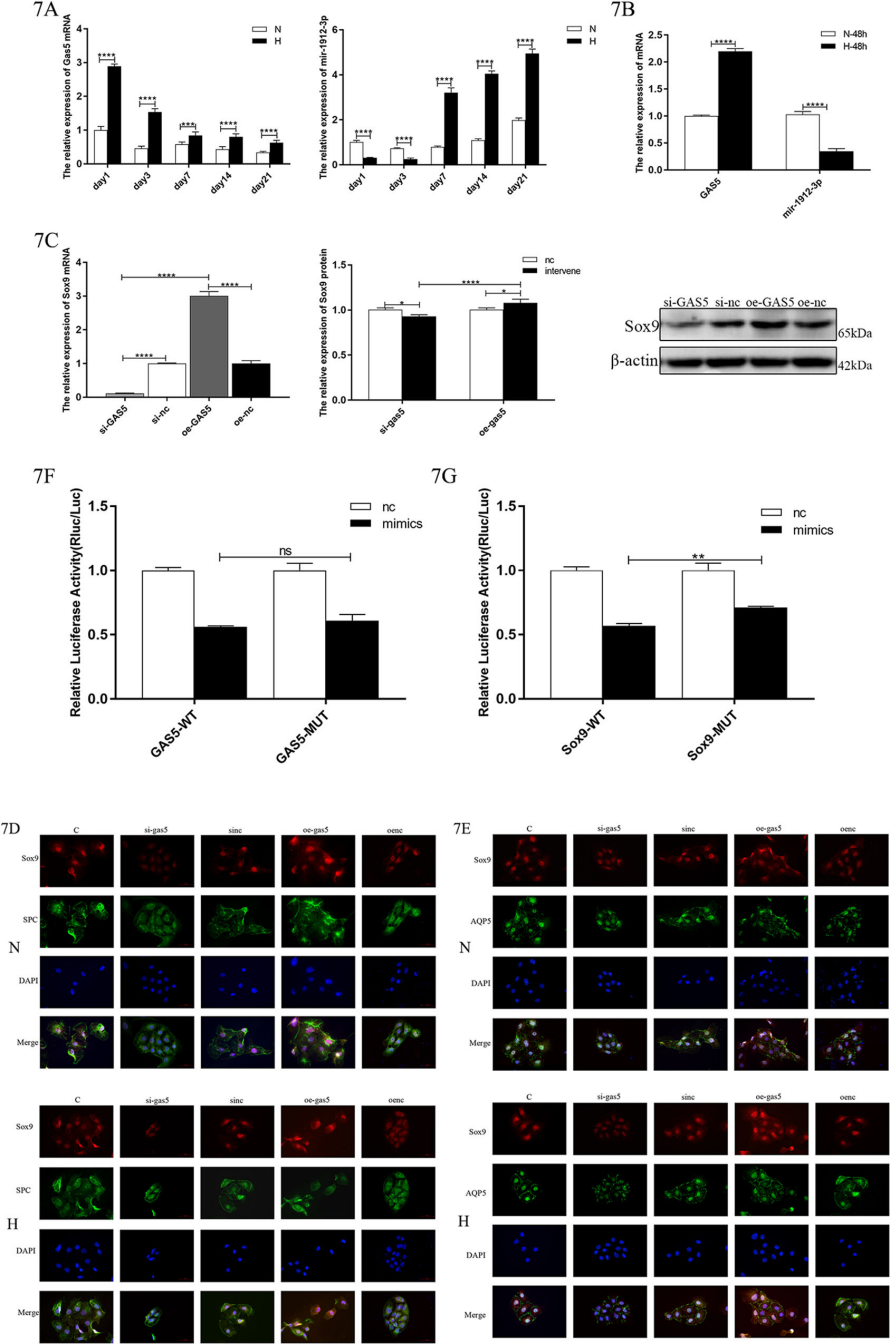
Changes in the expression of GAS5 and miR-1912-3p. **A** Expression level of GAS5 and miR-1912-3p in lung tissue (*n* = 6). **B** Expression level of GAS5 and miR-1912-3p in the in vitro cell model. **C** Relative levels of Sox9 mRNAs and protein after the GAS5 intervention.Target band intensity was normalized to β-actin (This blot is the cropped image). (***P < 0.005 and ****P < 0.001 compared with the control group). **D** Sox9(red)+ SPC (green) immunofluorescence double staining in primary cell model. **E** Sox9 (red) + AQP5 (green) immunofluorescence double staining in primary cell model. **F** Double luciferase report of GAS5 and miR-1912-3p. 7G: Double luciferase report of Sox9 and miR-1912-3p.

The PCR results of the animal model tissues showed that the expression of GAS5 in the model group was significantly increased and gradually decreased, which was positively correlated with the expression of Sox9. The expression of miR-1912-3p showed an overall upward trend that was negatively correlated with Sox9 (Fig. 7A). In vitro cell models, GAS expression increased, and miR-1912-3p expression decreased, which was consistent with the early expression of animal models (Fig. 7B).

We intervened with GAS5 in an in vitro cell model and detected the changes in the expression of Sox9 using PCR and western blotting (Fig. 7C). The results showed that the knockdown and overexpression of GAS5 could decrease or increase the expression of Sox9, respectively.

The extracted primary cells were subjected to the knockdown or overexpression of GAS5. The immunofluorescence double staining showed that the fluorescent intensity of Sox9 was weakened or enhanced, respectively (Fig. 7D, E).

The results of the dual-luciferase reporter gene assay showed that rno-miR-1912-3p could regulate the expression of luciferase with Gas5 (Fig. 7F, G). The overexpression of miR-1912-3p further reduced the luciferase activity of SOX9 WT-3ʹUTR. Additionally, the regulatory activity of Sox9 was restored after the predicted site mutation, but that of GAS5 was not significantly restored, indicating that rno-miR-1912-3p mimics may not have significant interactions with the predicted site of GAS5 but can bind to Sox9 through the predicted site.

## Discussion

Sox9 is an important regulatory factor in lung development. In the present study, we demonstrated for the first time that Sox9 can antagonize the overactivation of Wnt pathway in BPD and promote the differentiation of AEC-II, thereby improving the tissue morphology of lung tissue. In addition, the expression of Sox9 is regulated by lncRNA GAS5, which may be a target for therapeutic intervention.

Our study explored the protective role of Sox9 in BPD development and its effect on the Wnt pathway. The main pathological changes in BPD include alveolar simplification and pulmonary vascular dysplasia, which are the results of a complex interaction between changes in alveolar and vascular development, prenatal and postnatal pathogenic factors, and pulmonary repair processes [21]. In BPD, the excessive proliferation of AEC-II cells and their failure to differentiate into AEC-I cells are important reasons for insufficient damage repair. The Wnt/β-catenin signaling pathway has been found to play an important regulatory role in lung development, lung injury repair and regeneration, and lung disease progression [22], including non-small-cell lung cancer, chronic obstructive pulmonary disease, and idiopathic pulmonary fibrosis [23–26]. The canonical Wnt/β-catenin pathway is overactivated in BPD. Inhibiting the Wnt/β-catenin signaling pathway can reduce the proliferation of AEC-II cells, promote their differentiation into AEC-I cells [27], and also attenuate hyperoxia-induced lung injury in neonates [28]. Therefore, it is important to identify the factors in the relative signaling network that directly affect the key transcription factor β-catenin. As a member of the Sox family, Sox9 participates in biological processes through various signaling pathways, such as Notch, PI3K/AKT, Hippo, and Wnt, and has been found to exhibit tissue and spatiotemporal specificity [29–31]. Crosstalk between these different signaling pathways forms a complex signal-regulatory network [32, 33]. We found that the increased expression of Sox9 in the early stages of BPD could downregulate the expression of β-catenin and promote the differentiation of AEC-II cells into AEC-I, thereby alleviating the pathological changes in BPD. These findings contribute important nodes to the formation of the Wnt signaling pathway network.

Our study found that exogenous Sox9 improved alveolarization in animal models of BPD. This was achieved by promoting the proliferation of AEC-II cells and differentiation of AEC-I cells.Sox9 belongs to the SoxE subgroup and is located in the 3 Mb region of chromosome 17, which lacks other protein-coding genes. Sox9 is a dynamically expressed transcription factor and maintains stem cell function during lung development [34]. Rockich et al. showed that the expression of Sox9 during lung development maintains the undifferentiated state of distal lung progenitors is downregulated starting at embryonic day 16.5, concurrent with the onset of terminal differentiation of type 1 and type 2 alveolar cells. The removal of Sox9 leads to premature alveolar differentiation, whereas the addition of Sox9 prevents differentiation and inhibits the epithelial transition from columnar to squamous epithelium, which allows the adult alveoli to form [12]. However, another study on cultured lung organoids confirmed that the inactivation of Sox9 did not affect the differentiation of mouse alveolar cells or the ability of Sox9 to promote the recovery of damaged lung function [35]. In our study, lung of the animal models transfected with Sox9 showed improved alveolarization, an increased number of alveoli, and the number of SPC-labeled undifferentiated AEC-II cells was decreased, while the fluorescent expression of AQP5 was enhanced. This indicates that exogenous Sox9 can promote the proliferation and differentiation of AEC-II cells into AEC-I. Additional experiments revealed that its localization changed significantly.

We further found that the expression of Sox9 decreased in the nucleus and increased in the cytoplasm of the primary AEC-II cells induced with hyperoxia; therefore, we speculated that the overexpression of Sox9 in BPD may promote the differentiation of AEC-II cells through its nucleocytoplasmic translocation. The mechanisms by which the downregulation of the nuclear expression of Sox9 promotes AEC-II cell differentiation may be similar to those involved in the development of embryonic alveoli. The HMG domain of Sox9 contains two nuclear localization signal sequences and one leucine-rich nuclear export signal sequence and exhibits a high translocation rate between the nucleus and cytoplasm. Numerous studies have confirmed that the regulation of organogenesis by the Sox9 protein is not static but is a highly dynamic and complex process caused by the nucleocytoplasmic shuttling of Sox9. The nucleocytoplasmic translocation of Sox9 determines its function. During sex development, the balance between the nuclear import and export of Sox9 determines the level of transcriptionally active Sox9 in the nucleus. Its nuclear transfer further shifts the fate of undifferentiated somatic cells in primitive gonads away from the ovariogenesis pathway to testicular differentiation [36]. During kidney development, Sox9 is activated by nucleocytoplasmic translocation and promotes the proliferation and differentiation of renal tubular epithelial cells [37]. During corneal development and injury repair, Sox9 is synthesized in the cytoplasm of limbal stem or progenitor cells. When it translocates into the nucleus, Sox9 triggers the transformation of limbal epithelial progenitor cells into proliferating, transiently expanding cells and differentiation into the correct lineage. The regenerative potential is acquired through this process, which is achieved through the cooperation of Sox9 and Wnt/β-catenin signaling in a mutually inhibitory interaction [38]. The expression of Sox9 is increased during the formation of BPD, and the process of nuclear transfer occurs during the differentiation of AEC-II cells, which reflects that the transcriptional regulation of Sox9 plays an important role in the molecular mechanisms underlying BPD formation. However, the specific mechanisms underlying the changes in the nuclear and cytoplasmic distribution of β-catenin remain to be further elucidated.

Members of the canonical WNT signaling pathway play an important role in regulating the development of mammalian lung organs. The deletion of Wnt2/Wnt2b can lead to a reduction in the number of tracheal branches in the lungs and severe lung hypoplasia [39]. Wnt7b stimulates embryonic lung growth by coordinating increased epithelial and mesenchymal cell replication [8]. In mouse lung epithelial cells, the differentiation of alveolar epithelial cells with knocked-out β-catenin is hindered, and the lungs remain in the initial stage of tracheal formation [9]. In the process of lung development and injury repair, there is a crosstalk between the Wnt pathway and other signaling pathways. During the pseudoglandular stage of lung development, the fibroblast growth factor and WNT signaling pathways work together to support mesenchymal growth and coordinate epithelial morphogenesis [40]. Our previous studies also confirmed that the YAP pathway can regulate the expression of β-catenin in a manner that is independent of Wnt3a in BPD [41]. The SOX family of transcription factors has further emerged as a regulator of canonical Wnt/β-catenin signaling. SOX proteins can regulate β-catenin activity in various ways, including protein–protein interactions, the binding of SOX factors to the promoters of Wnt target genes, recruitment of co-repressors or co-activators, regulation of protein stability, and nuclear translocation [42]. Our study also revealed that not only can the overexpression of Sox9 in BPD reduce the overall expression level of β-catenin but also that the nucleocytoplasmic translocation of Sox9 can change the nucleocytoplasmic distribution of β-catenin by reducing its nuclear expression to inhibit the activation of the Wnt/β-catenin signaling pathway. β-Catenin is a membrane-bound protein that acts as a central mediator of transcriptional co-activators in canonical Wnt/β-catenin signaling [43]. β-Catenin is localized in the cytoplasm during lung development and is usually localized in the nuclei of undifferentiated primitive epithelial, differentiated alveolar epithelial, and adjacent stromal cells [44]. β-Catenin accumulates in the cytoplasm and translocates to the nucleus, thereby triggering Wnt target genes through T-cell factor and lymphoid enhancer factor proteins [43], which is the key mechanism in activating the canonical Wnt pathway. Our experiments demonstrate that the overexpression of Sox9 in BPD reduces the nuclear expression of β-catenin. Sox9 regulates β-catenin expression in many ways. Several studies have confirmed that Sox9 can directly bind to β-catenin, thereby interfering with the formation of the β-catenin/T-cell factor complex. Sox9 can also directly bind to β-catenin through the C-terminus. This results in the degradation of both proteins by the proteasome [45]. It has also been found that the N-terminus of Sox9, including the HMG domain, promotes the degradation of β-catenin, while the C-terminal transactivation domain inhibits the transcriptional activity of β-catenin without affecting its stability. Sox9 can also bind to components of the β-catenin destruction complex and relocate them to the nucleus. The nuclear localization of Sox9 is necessary and sufficient to promote the phosphorylation of β-catenin in the nucleus and its subsequent degradation [46]. A recent study confirmed that Sox9 could affect the stability of β-catenin and promote its degradation through the transcriptional activation of mastermind-like transcriptional activator 2 [39]. Sox9 overexpression in BPD further reduces the nuclear expression of β-catenin. This may be caused by the reduction of cytoplasmic β-catenin levels in the nucleus or because the nuclear expression of Sox9 promotes the increased degradation of β-catenin in the nucleus. The specific mechanisms underlying these actions remain to be further studied. However, these processes do not seem to promote the increased phosphorylation or degradation of β-catenin in the cytoplasm as the expression level of the β-catenin protein in the cytoplasm is increased.

The role of noncoding RNAs in development and disease has become a topic of interest in recent years. Many lncRNAs, including MALAT1, CASC2, H19, and NEF, have been confirmed to be associated with BPD. Our study confirmed that the expression of the lncRNA GAS5 is increased in BPD and can regulate the expression of Sox9. LncRNAs are RNAs whose transcripts exceed 200 nucleotides and are not translated into proteins. GAS5 plays multiple roles in lung diseases. For example, its expression is reduced in non-small-cell lung cancer, and its upregulation can inhibit tumor proliferation, invasion, and migration. GAS5, therefore, acts as a tumor suppressor by regulating various miRNAs, inhibiting epithelial–mesenchymal transition, activating signaling pathways, and inhibiting cell cycle progression and other pathways [47–51]. In acute lung injury, asthma, childhood pneumonia, and other diseases, GAS5 can affect the pyroptosis of bronchial epithelial cells, inhibit inflammatory responses and apoptosis, and promote the proliferation of airway smooth muscle cells [52]. GAS5 present in exosomes from patients with non-small-cell lung cancer may serve as an ideal serum-based non-invasive marker for identifying early-stage non-small-cell lung cancer [53]. The expression of GAS5 is increased in BPD and can regulate the expression of Sox9, thereby affecting AEC-II cell differentiation. We envisage that GAS5 may be used as an evaluation index in children with BPD to assess the severity and prognosis of the disease by collecting sputum, bronchoalveolar lavage fluid, or peripheral blood.

The mechanisms by which lncRNAs regulate gene expression are complex. They can directly bind to DNA or transcription factors to regulate gene expression at the transcriptional level. LncRNAs can also target mRNAs, miRNAs, or proteins and regulate their activity and stability to exert post-transcriptional effects or interfere with epigenetic cytoplasmic complexes, thereby inhibiting or activating gene expression [31, 54, 55]. We screened miRNAs that may have binding sites with GAS5 and Sox9 using the TargetScan database and other biological information networks. Eleven miRNAs, including miR-18a-5p, miR-200b-3p, miR-450b-5p, and miR-1912-3p, were identified. Unfortunately, the results showed that miR-1912-3p could not participate in the regulation of Sox9 by GAS5 through our predicted binding site

## Conclusion

In summary, our study found that increased nuclear entry of Sox9 in AEC-II cells with lung injury caused by hyperoxic exposure can downregulate the expression of β-catenin, enhance the ability of AEC-II cells to differentiate into AEC-I cells, and antagonize the alveolar disturbance caused by overactivation of Wnt pathway. In addition, the expression of Sox9 can be regulated by GAS5, which can be detected in exosomes, which may be a target for disease intervention. This can effectively improve the coordination and centrality of the network regulation of the cell damage repair signaling pathway. However, this study still has limitations. We have not yet revealed the mode of action of Sox9 and β-catenin in the nucleus, and the expression of GAS5 in body fluids and blood of BPD patients and its regulation of Sox9 needs further research.

## Materials and methods

### Animal model of bronchopulmonary dysplasia

All animal experiments were examined and approved by the Experimental Animal Ethics Committee of China Medical University. Adult Sprague–Dawley (SD) rats (8–10 weeks of age, weighing 200–250 g) were obtained from Changsheng Biotechnology Co., Ltd. (Liaoning, China). Animals were raised in specific-pathogen-free conditions and mated in cages with a male-to-female ratio of 4:1. Pregnant female SD rats were fed independently for 21–23 days and delivered naturally. Newborn SD rats were randomized to each group, and the number of rats in each group was 100. For the model group raised 85% O_2_ within 12 h after birth while the control group raised in 21% O_2_. Twenty young rats were randomly selected on days 1, 3, 7, 14, and 21, and the lung of rats was eviscerated. Formalin was instilled in the trachea to expand the alveoli as described [41].

### Cell culture

The rat AEC-II cell line RLE-6TN (ATCC, Manassas, VA, USA) was purchased and cultured in RPMI 1640 medium (Gibco, Grand Island, NY, USA) supplemented with 10% fetal bovine serum (Gibco, US) and 1% penicillin/streptomycin (HyClone, South Logan, UT, USA), and maintained at 37 °C, 5% CO2 humidified incubator. For the model group, the cell was incubated in a high-oxygen environment (85% O_2_, 5% CO_2_) for 48 h.

### Primary AEC-II cells culture

Lung tissues of newborn rats (at least 6 newborn rats at a time)within 12 h were minced on ice and predigested with 0.25% trypsin-EDTA (Gibco, Thermo Fisher Scientific) for 10 min. The same volume of DMEM/F12 medium (Gibco, Thermo Fisher Scientific) with 10% fetal bovine serum (Gibco, Thermo Fisher Scientific) was then added to terminate the predigestion, after which the mixture was discarded. Trypsin-EDTA (0.25%) was added to the lung tissue to digest for 20 min, and an equal volume of DMEM/F12 medium was added to terminate the digestion and collect the digested liquid. Afterward, 0.1% collagenase type I (Gibco, Thermo Fisher Scientific) was added to the lung tissue to digest the cells for 30 min, and an equal volume of DMEM/F12 medium was added to terminate the digestion and collect the digested liquid. The alternate digestion process was repeated three times. The harvested cell suspension was filtered through a 200-mesh cell strainer and centrifuged (4 °C, 5 min, and 200 × *g*). The cell pellet was resuspended in DMEM/F12 medium with 10% fetal bovine serum and 1% penicillin-streptomycin (HyClone) and incubated in a 5% CO_2_ incubator at 37 °C. The fibroblasts were removed and purified using the differential attachment method. Following 12 h of culture, in order to demonstrate the effectiveness and reliability of the isolating and purifying method used in this study, the characteristics of isolated and purified AEC II cells from the lungs were confirmed by a light microscope and immunofluorescence staining: the cells were found to be round, oval, or polygonal in shape under a light microscope and showed island-like adherent growthpurity of AECIIs (>90%) were measured with a surfactant protein C (SPC), a unique AEC II biomarker. The activity of AECIIs (>95%) were measured using trypan blue staining for 3 min at 37 °C(C0011‑1, Beyotime Institute of Biotechnology, Inc.). Then cells for the model group were incubated in a high-oxygen environment (85% O_2_, 5% CO_2_), and collected after 24, 48, or 72 h for subsequent experiments.

### Cell transfection

The siRNAs (GAS5 (Norway rat) si-RNA, Sox9 (Norway rat) si-RNA), overexpression plasmids (pZdonor-CMV-Gas5 (rat, NR-002704)-SV40 promoter-neo, pZdonor-CMV-Sox9 (rat, NM-080403)-SV40 promoter-neo), and corresponding negative controls (si-nc Negative control, oe-nc pZdonor-CMV-MCS-SV40 promoter-neo) of GAS5 and Sox9 were obtained from SyngenTech (Beijing, China). The cells were seeded in 6- or 24-well plates for 12 h before the experiment. Transfection was performed using the Polyplus jetPRIME transfection kit (Polyplus Transfection, France) according to the manufacturer’s instructions.

MiR-1912-3p mimics and inhibitors and the corresponding negative controls were purchased from Guangzhou RiboBio Co., Ltd. Transfection was performed using the RiboFECTTMCP transfection reagent (RiboBio Co. Ltd., Guangzhou, China) according to the manufacturer’s protocol.

### Animal transfection

Rats were suspended at 45° by the upper teeth on a rodent dosing board, and the trachea was visualized using a fiber optic stylet connected to an endotracheal tube (Biolite small animal intubation system, Kent Scientific Corp, USA). The trachea was intubated, and oe-Sox9 plasmid(1 μg/g) in 50 μl saline was delivered, followed by 200 μl of air [56]. The rats were injected once every other day from the fifth day of high oxygen until the 14th day, and the lungs were taken from the rats, and their lungs were harvested and subsequently frozen in a −80 °C deep-frozen refrigerator for later analysis of tissue sections.

### Hematoxylin & eosin (H&E) staining

H&E staining was performed as in our previous study [41]. Fixed lungs with formalin were embedded in paraffin and sectioned to 3 μm, followed by staining with hematoxylin-eosin stain.

### Immunofluorescence staining

The lung paraffin sections were dewaxed, hydrated, antigen-repaired, and circled. The paraffin-embedded sections or primary cell slides were sealed with 10% goat serum at room temperature (22–24 °C) for 1 h, then away from light at 4 °C overnight with antibodies:anti-sox9 (1:100, Santa Cruz), anti-β-catenin (1:200, Proteintech), anti-SPC (1:100, Proteintech), and anti-AQP5 (1:100, Proteintech). Next, the goat anti-rabbit or goat anti-mouse secondary antibody (1:200, Proteintech) was used to incubate for 2 h. After DAPI was used to stain the cell nucleus, a fluorescence microscope (E800, Nikon, Japan) was used to observe protein expression.

### RNA extraction and quantitative real-time PCR

The total RNA of cells or tissues was extracted usingTransZol reagent (TransGen, Beijing, China), and cDNA was synthesized using TransScript.One-Step gDNA Removal and cDNA Synthesis SuperMix(TransGen). Quantitative real-time PCR was performed using PerfectStart Green qPCR SuperMix (TransGen). The expression levels of target genes were uniformly normalized to Actin. All primers used in this study were listed in Supplementary Table 1.

### Western blotting

Cells or tissue were lysed in RIPA Lysis Buffer (TransGen Biotech, Beijing, China) contained with 1% Phenylmethanesulfonyl fluoride (TransGen Biotech). Proteins (30 μg/lane) were separated on 10% SDS-polyacrylamide gradient gels and transferred onto PVDF membranes. The protein-free rapid sealing fluid (Epizyme, Shanghai, China) was used to block non-specific binding, and membranes were probed with primary antibodies in 4 °C for 12 h(anti-SOX9 (1:500, Santa Cruz, Santa Clara, CA, USA), anti-β-catenin (1:10 000, Proteintech, Shanghai, China), anti-SPC (1:500, Proteintech), anti-AQP5 (1:500, Santa, USA), and anti-β-actin (1:20 000, Proteintech)), followed by incubation with anti-rabbit-HRP(1:5000; Proteintech) or anti-mouse-HRP (1:5000; Proteintech) in 37 °C for 2 h. β-actin was selected as the internal reference. The protein bands were visualized with the enhanced chemiluminescence western blotting detection system (Millipore, Billerica, MA, USA).

### Luciferase reporter assay

Wild-type (WT) and mutant target fragments of GAS5 and Sox9 were constructed, and their corresponding vectors (Wt-GAS5, Mut-GAS5, Wt-Sox9, and Mut-Sox9) were established. miR-1912-3p and miR-NC were co-transfected into HEK293T cells. After transfection for 48 h, luciferase activity was determined using a dual-luciferase reporter assay system (E1960, Promega, Madison, WI, USA) according to the manufacturer’s instructions.

### Flow cytometry detect AEC-II cell differentiation

The primary cells were sealed with 5% bovine serum albumin solution at room temperature for 1 h. The AEC-I cells were fluorescently labeled using the AQP5 antibody (Biorbyt, Cambridge, England), and the AEC-II cells were fluorescently labeled with anti-rabbit IgG (H + L) F(AB’)2 Fragment (PE Conjugate) (CST, KS, USA) and Furin AB (Novus Biologicals, CO, USA). The fluorescently labeled cell suspensions were assessed using flow cytometry (BD, Franklin Lake, NJ, USA).

### Statistical analysis

All analyses were performed using GraphPad Prism 7 software. Data were expressed as mean ± standard deviation (SD). The differences in the data were analyzed by one‑way or two‑way analysis of variance. Unless otherwise specified, all experiments involved at least 6 rats and were representative of at least three independent experiments. A confidence interval of 95% was used for all statistical tests, and *P* < 0.05 was regarded to be statistically significant.

### Supplementary information

Supplementary materials

Supplementary table

## Data Availability

The datasets used and/or analyzed during the current study are available from the corresponding author upon reasonable request.
