# Supplementary material for: Role of Sox9 in BPD and its effects on the Wnt/β-catenin pathway and AEC-II differentiation
Source: Cell Death Discov. 2024 Jan 11;10:20. doi: 10.1038/s41420-023-01795-2 (PMC10784471; doi:10.1038/s41420-023-01795-2)

1. Original image

Fig 1E

Target band of AQP5 in animal model:

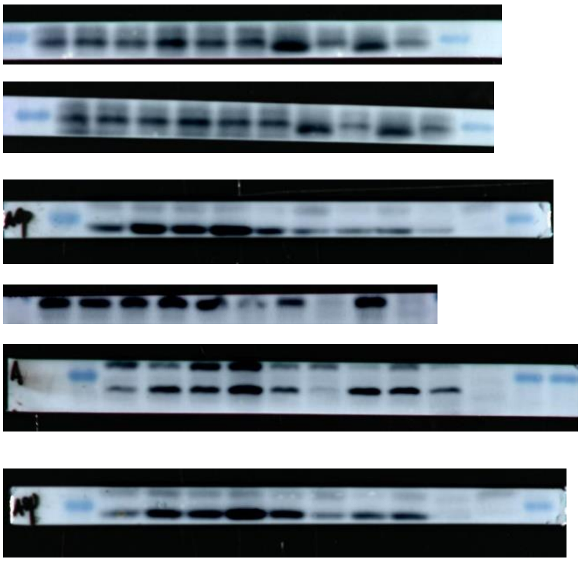

Target band of SPC in animal model:

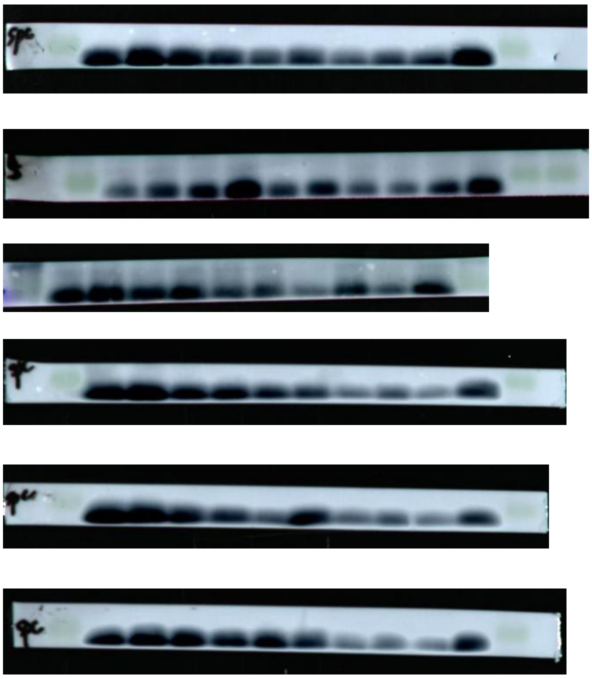

Target band of Sox9 in animal model:

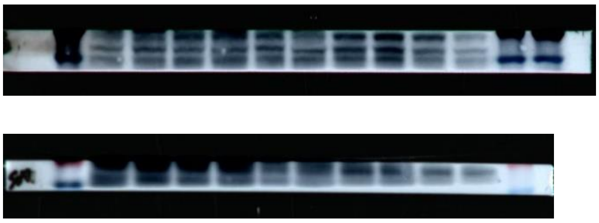

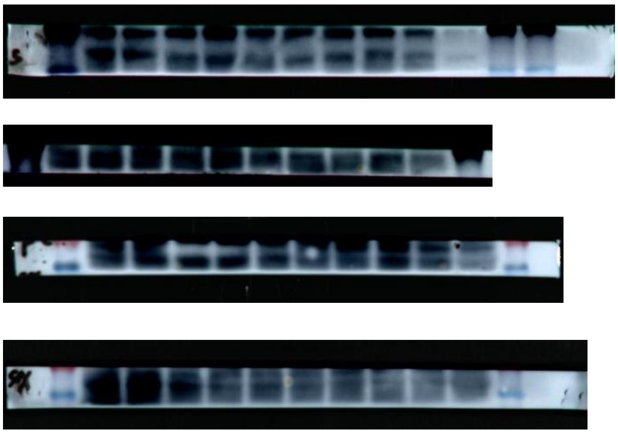

Fig 1G

Target band of Sox9 in the in vitro cell model

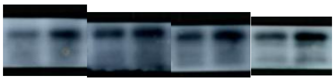

Target band of SPC in the in vitro cell model

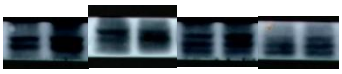

Fig 2D

Total protein and plasma protein target bands of Sox9 in the in vitro cell model

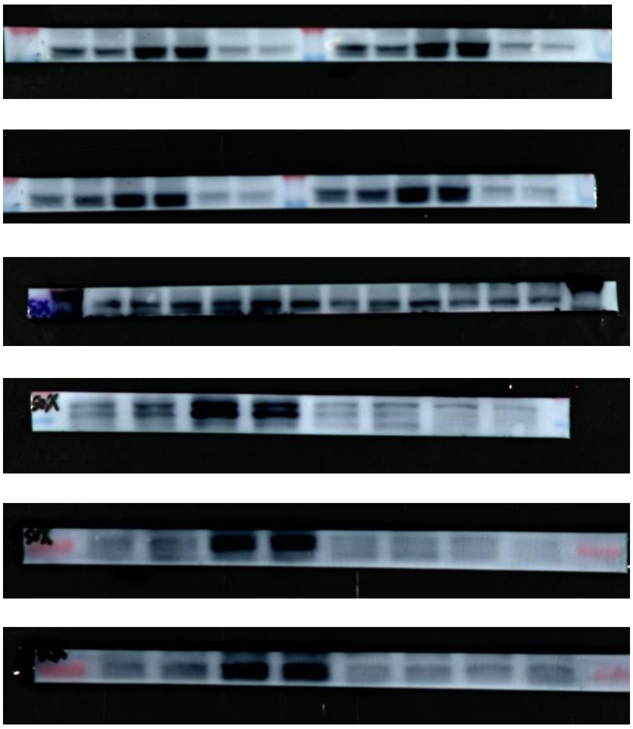

Fig 4C

Target band of Sox9 after Sox9 intervention

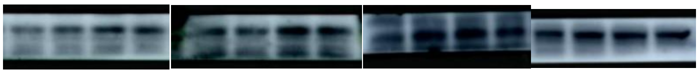

Fig 5C

Target band of β-catenin in animal model

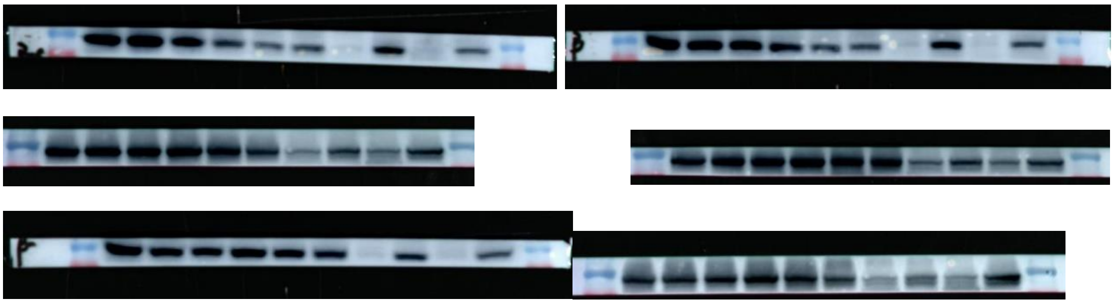

Fig 5F

Target band of β-catenin in the in vitro cell model after Sox9 intervention

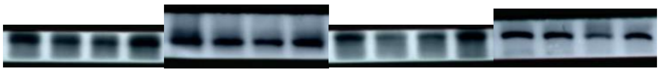

Fig 6B

The total protein and plasma protein target band of β-catenin in the in vitro cell model

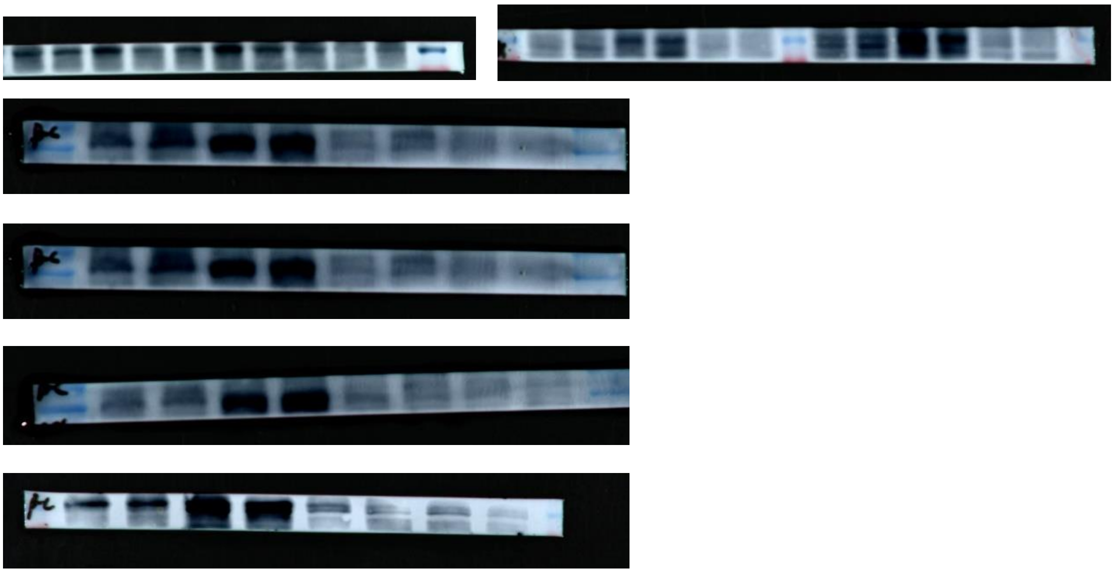

Fig 6E

The total protein and plasma protein target band of β-catenin in the hyperoxia cell model after the overexpression of Sox9

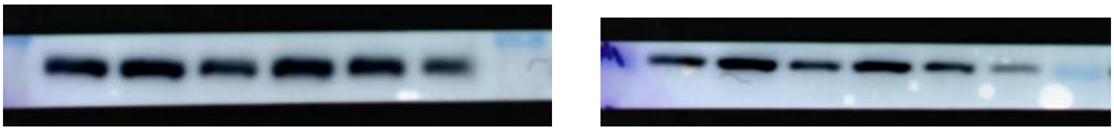

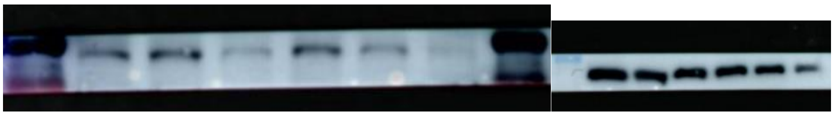

The total protein and plasma protein target band of Sox9 in the hyperoxia cell model after the overexpression of Sox9

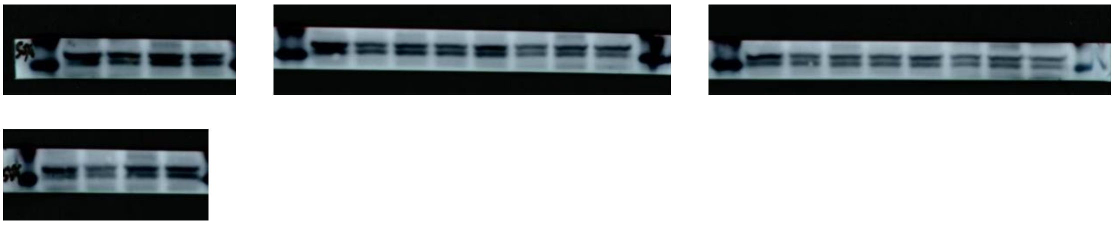

Fig 7C

Target band of Sox9 after the GAS5 intervention

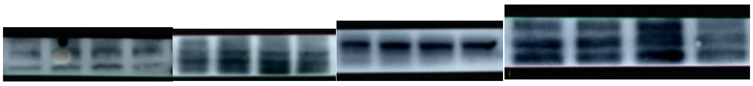

2.Efficacy of knockdown and overexpression

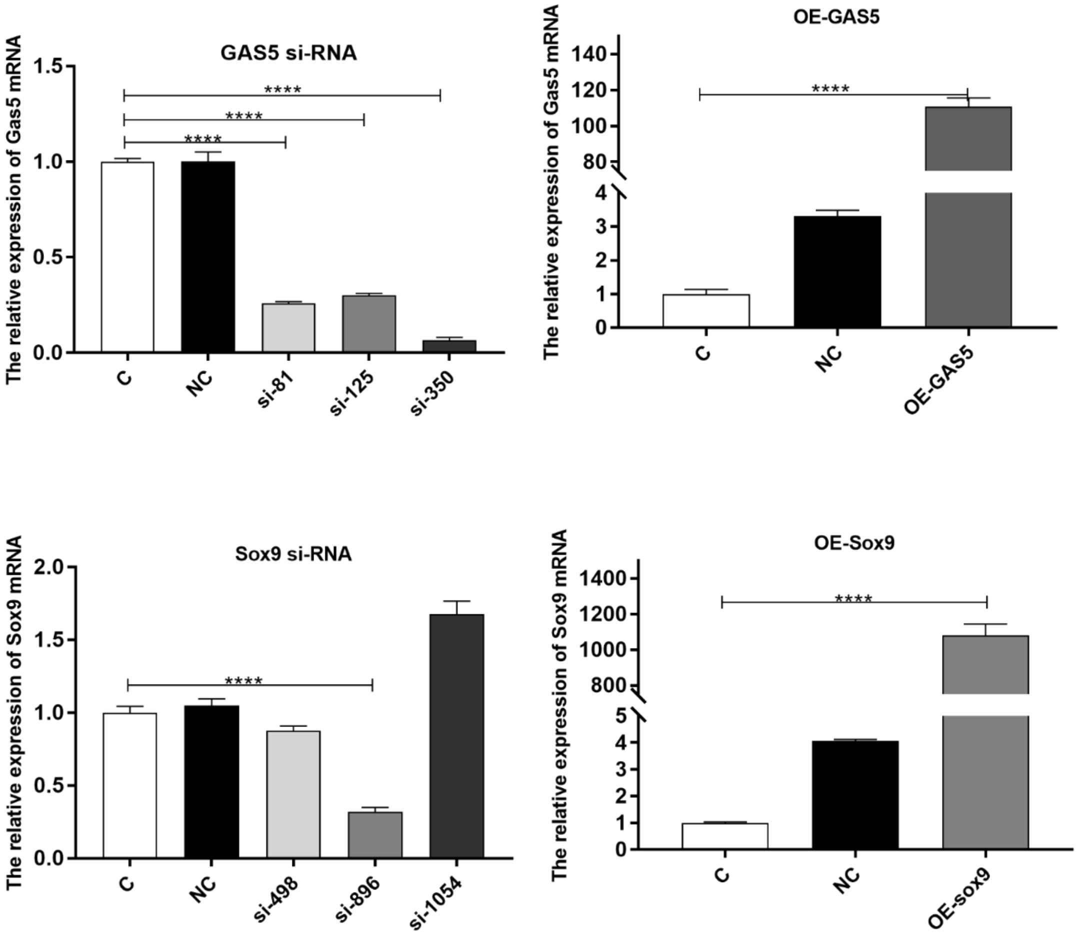

Supplement: Supplementary file 1 — Supplementary materials [file 41420_2023_1795_MOESM1_ESM.pdf]
