# Supplementary material for: Role of Sox9 in BPD and its effects on the Wnt/β-catenin pathway and AEC-II differentiation
Source: Cell Death Discov. 2024 Jan 11;10:20. doi: 10.1038/s41420-023-01795-2 (PMC10784471; doi:10.1038/s41420-023-01795-2)
Supplement: Supplementary file 2 — Supplementary table [file 41420_2023_1795_MOESM2_ESM.docx]

Table 1 primer sequences

| primer | primer sequences（5’to3’） |
| --- | --- |
| GAS5-F | GCAAGCTCCACACAAGGTCCTTC |
| GAS5-R | TGTTCAAGCATCCATCCAGTCACC |
| Sox9-F | ACTCCGGCTCCTACTACAGC |
| Sox9-R | CTGCGCTGGGTTCATGTAGG |
| SPC-F | CCCAGGAGCCAGTTTCGCATTC |
| SPC-R | GACGACAAGGACTACCACCACAAC |
| AQP5-F | CCATGAACCCAGCCCGATCTTTC |
| AQP5-R | CCCTACCCAGAAGACCCAGTGAG |
| β- actin-F | GGAGATTACTGCCCTGGCTCCTA |
| β- actin-R | GACTCATCGTACTCCTGCTTGCTG |
| miR-1912-3p primer was synthesized by Guangzhou RiboBio Co., Ltd, and the sequence was not provided due to technical patents involved. | |
